# Immune checkpoint inhibitor-related intestinal pseudo-obstruction and multisystem immune-related adverse events in a post-gastrectomy patient: a case report and literature review

**DOI:** 10.3389/fonc.2026.1904273

**Published:** 2026-08-31

**Authors:** Zhening Liu, Chao Wang, Yulei Shen, Yanfen Shi, Jianghui Duan, Huijing Dong, Yangbo Xu, Yumin Zheng, Huijuan Cui

**Affiliations:** 1 Graduate School, Beijing University of Chinese Medicine, Beijing, China; 2 Department of Integrative Oncology, China-Japan Friendship Hospital, Beijing, China; 3 Department of Pathology, China-Japan Friendship Hospital, Beijing, China; 4 Department of Radiology, China-Japan Friendship Hospital, Beijing, China; 5 Department of Oncology, Ningbo Municipal Hospital of Traditional Chinese Medicine, Ningbo, Zhejiang, China

**Keywords:** case report, gastric cancer, immune checkpoint inhibitors, intestinal pseudo-obstruction, nivolumab

## Abstract

**Background:**

Immune checkpoint inhibitors are increasingly recognized to be associated with multisystem immune-related adverse events. Cases presenting predominantly with intestinal pseudo-obstruction (IPO) are rare, prone to misdiagnosis, and may indicate broader immune-mediated toxicity.

**Case presentation:**

We report the case of a patient with gastric cancer who developed intestinal pseudo-obstruction and multisystem immune toxicity during postoperative adjuvant chemotherapy plus nivolumab. The patient’s symptoms improved rapidly after glucocorticoid treatment.

**Conclusion:**

Immune checkpoint inhibitor-related intestinal pseudo-obstruction (irIPO) should be recognized early and diagnosed on the basis of a comprehensive clinical assessment. Differential diagnosis, multidisciplinary collaboration, and active screening for additional organ involvement are essential. Early glucocorticoid treatment may improve clinical outcomes.

## 1 Introduction

Immune checkpoint inhibitors (ICIs) restore and enhance T-cell-mediated antitumor immune responses by blocking immunosuppressive pathways such as programmed cell death protein 1 (PD-1)/programmed death-ligand 1 (PD-L1) and cytotoxic T-lymphocyte-associated antigen 4 (CTLA-4), and are now widely used to treat various solid tumors (1, 2). Nevertheless, the broad immune activation induced by ICIs may disrupt immune tolerance and lead to immune-related adverse events (irAEs) involving multiple organ systems, including the skin, lungs, liver, endocrine system, nervous system, and gastrointestinal tract (3, 4). Gastrointestinal irAEs most commonly manifest as immune checkpoint inhibitor-associated diarrhea and colitis (5), whereas immune checkpoint inhibitor-related intestinal pseudo-obstruction (irIPO) is a rare but increasingly recognized irAE.

IPO is a gastrointestinal motility disorder caused by dysfunction of the intestinal smooth muscle or enteric nervous system. It is characterized by symptoms and imaging findings suggestive of bowel obstruction in the absence of a demonstrable mechanical obstruction (6). As a rare but serious irAE during immunotherapy, irIPO can be mistaken for mechanical intestinal obstruction or tumor progression, potentially resulting in inappropriate management. In severe cases, it may be life-threatening. Moreover, because irIPO is not included in current immune toxicity management guidelines and no specific biomarkers are available, its diagnosis and management remain challenging.

We report a post-gastrectomy patient with gastric cancer who developed intestinal pseudo-obstruction and multisystem irAEs during adjuvant chemotherapy plus nivolumab, and discuss the diagnostic approach, potential mechanisms, and management implications in the context of the existing literature.

## 2 Case presentation

A 65-year-old man presented with intermittent upper abdominal discomfort without an obvious precipitating factor in June 2024. On July 22, 2024, he underwent laparoscopic radical distal gastrectomy with gastrojejunostomy (Billroth II reconstruction) at an outside hospital. Postoperative pathology revealed poorly differentiated adenocarcinoma with neuroendocrine differentiation, with areas showing signet-ring cell carcinoma, poorly cohesive carcinoma, and a minor mucinous adenocarcinoma component. The postoperative pathological stage was pT4aN3bM0, stage IIIC. His medical history was notable for a 15-year history of hypertension and a 30-pack-year smoking history (20 cigarettes/day), although he had quit smoking 5 years earlier. He had no history of autoimmune disease, chronic intestinal motility disorders, or opioid use.

**Figure 1:**
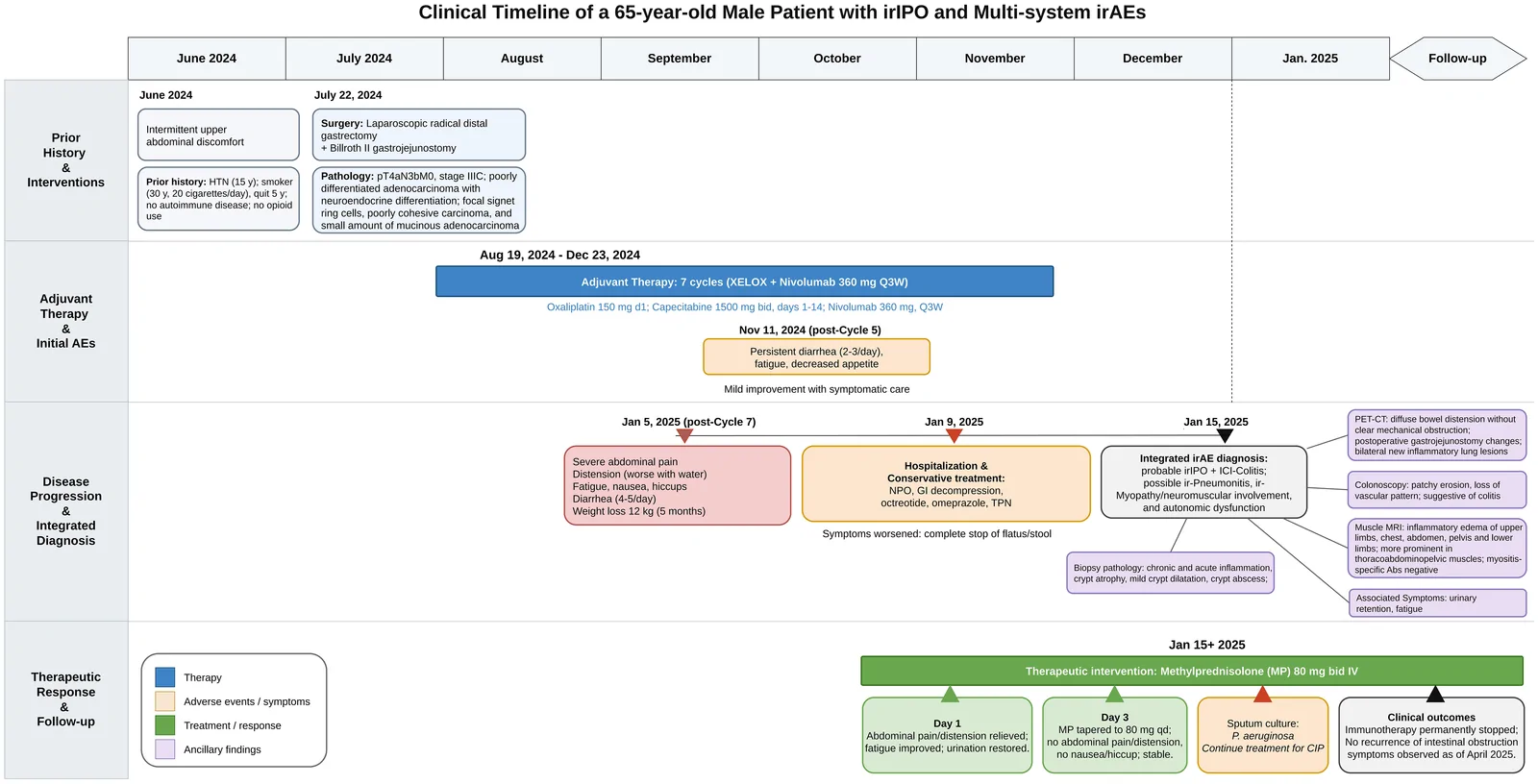
Clinical timeline showing the patient’s treatment course, onset and diagnosis of irIPO and multisystem irAEs, corticosteroid therapy, treatment response, and follow-up. Abbreviations: ICI, immune checkpoint inhibitor; irAEs, immune-related adverse events; irIPO, immune checkpoint inhibitor-related intestinal pseudo-obstruction; MRI, magnetic resonance imaging; PET/CT, positron emission tomography/computed tomography; TPN, total parenteral nutrition; XELOX, capecitabine plus oxaliplatin.

From August 19, 2024, to December 23, 2024, the patient received seven cycles of postoperative adjuvant XELOX combined with nivolumab. The regimen consisted of oxaliplatin 150 mg on day 1, capecitabine 1,500 mg twice daily on days 1–14, and nivolumab 360 mg every 3 weeks. On November 11, 2024, shortly after the fifth cycle, he developed persistent diarrhea (2–3 episodes/day), fatigue, and decreased appetite. These symptoms improved slightly after symptomatic treatment. On January 5, 2025, he developed persistent abdominal pain and abdominal distension, which worsened after water intake, accompanied by fatigue, nausea, hiccups, and increased diarrhea (4–5 episodes/day). Over the preceding 5 months, he had lost approximately 12 kg. Detailed laboratory examinations and the therapeutic course are presented in **Table 1** and **Figure 1**.

**Table 1:** Laboratory examinations.

Laboratory tests	Jan 2025	Reference range	Unit
White blood cell count	3.19	3.5–9.5	10^9^/L
Red blood cell count	3.05	4.3–5.8	10^12^/L
Platelet count	111	125–350	10^9^/L
C-reactive protein	17.42	< 10	mg/L
Procalcitonin	<0.20	< 0.5	ng/mL
Na	124.5	137–147	mmol/L
K	3.9	3.5–5.3	mmol/L
Ca	2.02	2.11–2.52	mmol/L
Aspartate aminotransferase	19	0–40	U/L
Alanine aminotransferase	8	9–50	U/L
Lactate dehydrogenase	170	100–250	U/L
Total bilirubin	21.78	≤ 23.00	μmol/L
Creatinine	42.7	53–106	μmol/L
Estimated glomerular filtration rate (eGFR)	114.38		mL/min/1.73m^2^
Albumin	29.8	40.0–55.0	g/L
Prealbumin	89.4	200–430	mg/L
Creatine kinase	27	26–200	U/L
Cardiac troponin I	0.0081	< 0.0198	ng/mL
D-dimer, quantitative	2.67	0–0.55	mg/L
Interleukin-6	36.39	< 5.40	pg/mL
Interleukin-8	33.74	< 20.60	pg/mL
Interleukin-1β	18.10	< 12.4	pg/mL
Thyroid-stimulating hormone	1.160	0.27–4.20	μIU/mL
Triiodothyronine	0.619	0.8–2.0	ng/mL
Thyroxine	7.220	5.1–14.1	μg/dL
Antinuclear antibody (ANA)	Negative		
Myositis antibodies	Negative		
HMGCR antibody	Negative		
Systemic sclerosis antibodies	Negative		

**Figure 2:**
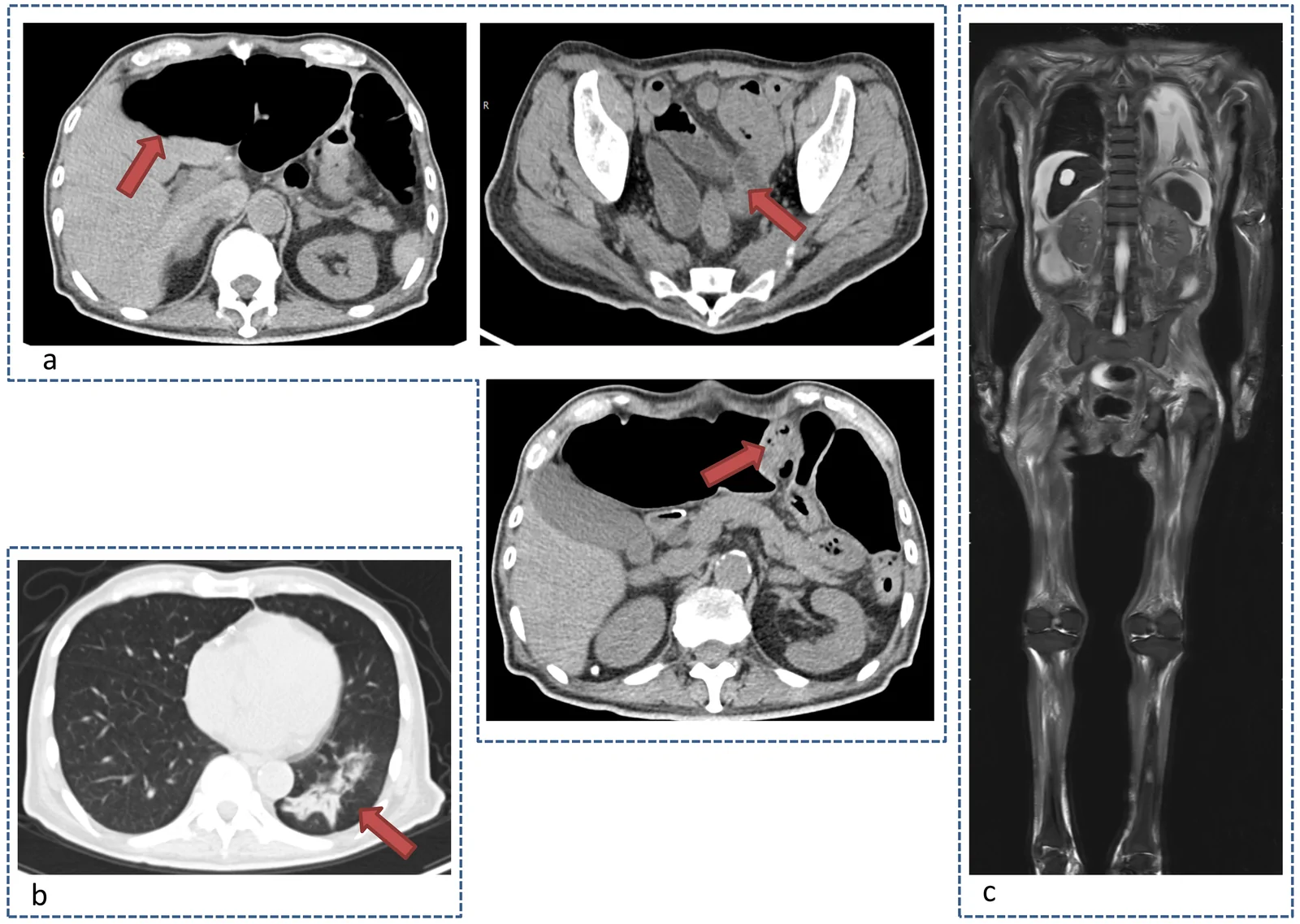
PET/CT and MRI findings showing intestinal pseudo-obstruction and multisystem inflammatory involvement. **(a)** PET/CT showed diffuse gaseous distension of the bowel loops, without a definite site of mechanical obstruction, mass lesion, or fixed transition point. Postoperative changes related to gastrojejunostomy were noted. **(b)** PET/CT showed patchy inflammatory exudative opacities in the left lung. **(c)** Whole-body muscle MRI showed multifocal hyperintense signals in the trunk, pelvic, and lower-limb muscles, accompanied by adjacent soft-tissue edema.

On January 9, 2025, PET/CT showed diffuse bowel dilatation with gaseous accumulation, suggestive of bowel obstruction, but no definite transition point or focal obstructing lesion was identified. Postoperative changes at the gastrojejunal anastomosis and newly developed inflammatory lesions in the left lung were also noted (**Figures 2a, b**). Conservative management was initiated for the obstructive symptoms, including fasting, gastrointestinal decompression, octreotide, omeprazole, and total parenteral nutrition. However, his symptoms did not improve, and he subsequently developed complete cessation of flatus and bowel movements.

**Figure 3:**
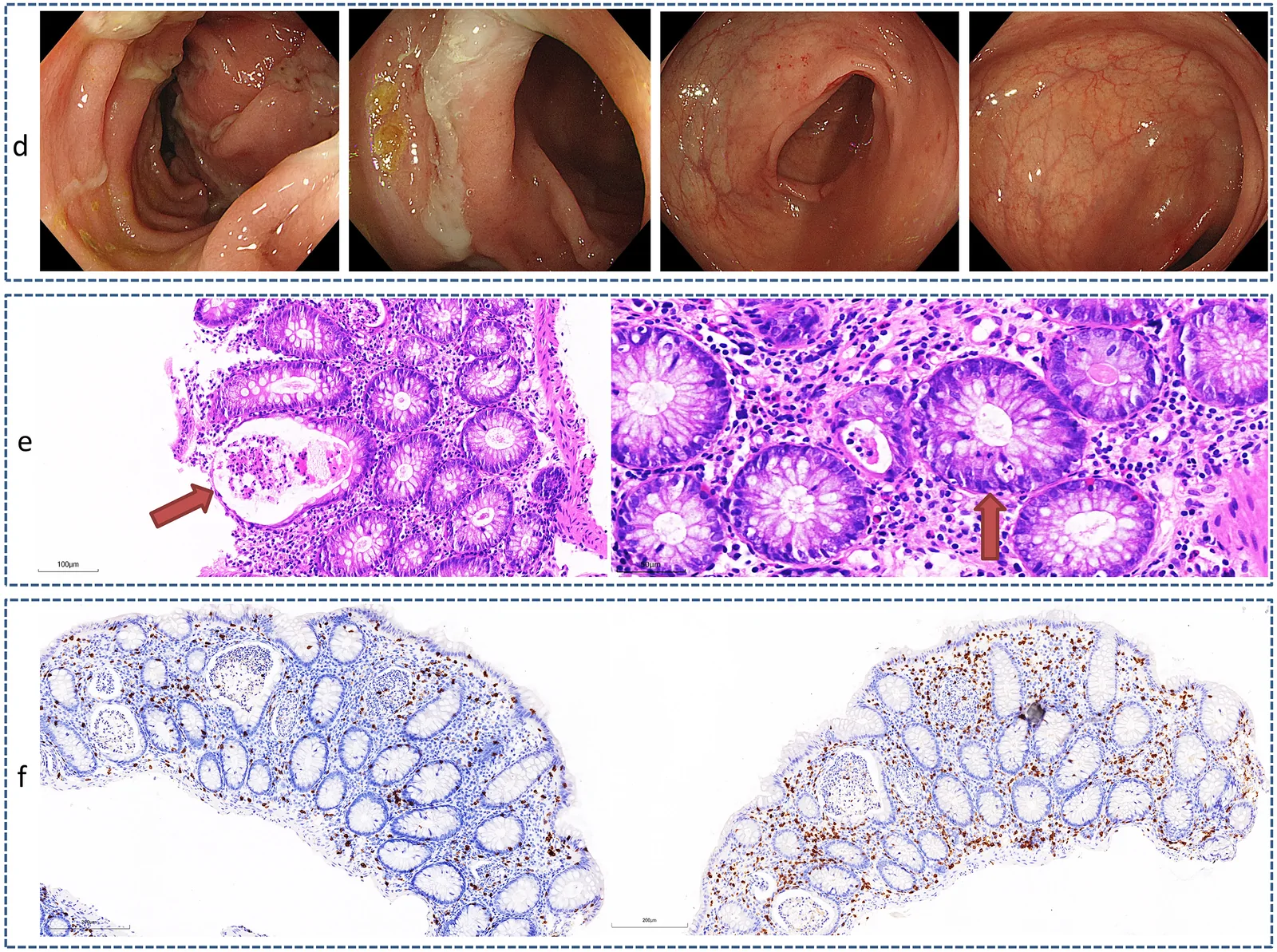
Colonoscopy and histopathological findings showing T-cell-mediated inflammatory injury of the colonic mucosa. **(d)** Colonoscopy showed hyperemic and edematous colonic mucosa with superficial erosions and loss of the normal vascular pattern. **(e)** Hematoxylin and eosin staining showed crypt abscesses, crypt atrophy, epithelial apoptosis, and inflammatory cell infiltration in the lamina propria. Scale bars: 100 μm and 50 μm. **(f)** Immunohistochemical staining showed increased CD3-positive and CD8-positive T lymphocytes in the colonic mucosa. Scale bar: 200 μm.

To further clarify the etiology, colonoscopy was performed on January 15, 2025. It revealed patchy erosions of the colonic mucosa approximately 40 cm from the anal verge, with loss of the vascular pattern, consistent with colitis (**Figure 3d**). Histopathological examination of biopsy specimens demonstrated moderate acute and chronic inflammatory cell infiltration with crypt atrophy, mild dilatation of some crypts, and crypt abscess formation (**Figure 3e**). Immunohistochemistry demonstrated infiltration of CD3+ and CD8+ T cells within the mucosa (**Figure 3f**). In the appropriate clinical context, these findings supported the diagnosis of immune checkpoint inhibitor-associated colitis (ICI-colitis). A clinical diagnosis of irIPO complicated by ICI-colitis was established on the basis of several converging findings. These included the temporal association with nivolumab exposure, diffuse bowel dilatation in the absence of mechanical obstruction, colonoscopic and histopathological evidence of colitis, and a rapid response to glucocorticoids despite limited benefit from conventional treatment.

In addition to gastrointestinal manifestations, the patient developed several extraintestinal findings suggestive of possible multisystem immune-related toxicity. He developed fatigue, chest tightness, dyspnea, and urinary retention. PET/CT demonstrated diffuse bronchitic and bronchiolitic changes with newly developed inflammatory lesions in the left lung. To further evaluate possible neuromuscular involvement, whole-body muscle MRI was performed. It showed varying degrees of increased STIR signal intensity in the muscles and subcutaneous fat of both upper limbs, chest, abdomen, pelvis, and lower limbs, suggestive of inflammatory exudative changes. Inflammatory edema of the thoracic, abdominal, and pelvic muscles was more prominent than that in the extremities (**Figure 2c**). The patient did not report myalgia, and both the myositis-specific antibody panel and anti-HMGCR antibody were negative. Therefore, the available evidence was insufficient to establish a definitive diagnosis of immune-related myositis, although immune-mediated myopathic or neuromuscular involvement was suspected.

In this case, given the patient’s severe pseudo-obstruction symptoms, inadequate response to conservative treatment, concurrent ICI-colitis, and suspected extraintestinal immune involvement, an individualized, intensified corticosteroid regimen was initiated. Methylprednisolone was administered at an initial dose of 160 mg/day (3 mg/kg/day) on the first day. A proton pump inhibitor (PPI) was co-administered for gastric mucosal protection, trimethoprim-sulfamethoxazole (TMP-SMX) was prescribed for infection prophylaxis, and blood glucose levels were closely monitored. Within 24 hours of treatment, the abdominal pain and distension significantly improved, fatigue was alleviated, and urinary retention resolved. After 3 days of high-dose therapy, following continuous clinical improvement, the dose of methylprednisolone was reduced to 80 mg/day (1.5 mg/kg/day). The patient no longer had abdominal pain or distension and no nausea or hiccups. On auscultation, bowel sounds remained hypoactive, with only 1–2 sounds per minute, but fatigue had improved further. Follow-up chest CT showed partial improvement of the lesions. Sputum culture yielded Pseudomonas aeruginosa, raising concern for immune-related pneumonitis complicated by superimposed pulmonary infection. The patient subsequently received treatment for pneumonia, and immunotherapy was permanently discontinued. After discharge, oral prednisone was started at 50 mg/day and gradually tapered over 8 weeks. At follow-up through April 2025, no recurrence of obstructive gastrointestinal symptoms was observed.

## 3 Discussion

With the increasing use of immune checkpoint inhibitors (ICIs) in solid tumors, the clinical spectrum of gastrointestinal immune-related adverse events has expanded. In addition to relatively common manifestations such as ICI-colitis and diarrhea, ICI-related gastrointestinal dysmotility has increasingly been reported in recent years. Although this complication appears to be uncommon and has mainly been described in case reports or small case series, it can have severe clinical consequences. A case series and systematic review including 18 previously reported cases of gastrointestinal dysmotility following ICI therapy found that the mean time to onset was approximately 13.6 weeks after ICI exposure, the rate of gastrointestinal recovery was only 38.5%, and the mortality rate was as high as 47.0% (7). These findings indicate that ICI-related gastrointestinal dysmotility is a serious toxicity that warrants recognition as a distinct clinical entity. In addition to evidence from case reports and small-scale reviews, real-world pharmacovigilance data also support an association between ICIs and bowel obstruction-related events. Gao et al. (8), using the FAERS database, analyzed 105,001 reports of ICI-related adverse events from 2011 to 2020 and identified 245 cases of intestinal obstruction, demonstrating a significant signal association between all ICIs and intestinal obstruction (ROR 4.27, 95% CI 3.75–4.85), with PD-1 inhibitors accounting for the largest number of reports. These findings suggest that, although such events are rare, they represent a real-world safety concern warranting clinical attention.

However, existing studies remain limited in their discussion of complex clinical scenarios. In patients with gastrointestinal malignancies after surgery, recognition of irIPO may be even more challenging. In such patients, multiple confounding factors, including postoperative adhesions, tumor recurrence, peritoneal disease, and infection, can easily bias clinicians toward a diagnosis of mechanical intestinal obstruction, thereby increasing the risk of misdiagnosis. In addition, previous reports have largely summarized these events from the broader perspectives of gastrointestinal dysmotility or autonomic neuropathy, whereas relatively little attention has been paid to cases in which irIPO is the predominant clinical problem and is accompanied by multisystem manifestations such as colitis, urinary retention, and muscle involvement. This case is notable because it shows that irIPO can occur after gastrectomy for gastric cancer and suggests that it may represent a gastrointestinal manifestation of broader multisystem neuroimmune toxicity. The strength of evidence for immune-related involvement, however, varied across organ systems, and some extraintestinal manifestations may also have had alternative or concurrent explanations. By presenting the diagnostic and therapeutic process in detail, this case provides a more clinically relevant framework for recognition and decision-making in real-world practice.

### 3.1 Differential diagnosis of irIPO

At present, no widely available diagnostic gold standard for irIPO exists. Although biopsy of the myenteric plexus may provide evidence approaching pathological confirmation, it is rarely performed in clinical practice because of its invasive nature. Therefore, in most cases, the diagnosis relies primarily on comprehensive clinical judgment, including the absence of evidence of mechanical obstruction, exclusion of common secondary causes, a temporal association with ICI exposure, and a supportive response to immunosuppressive therapy (9). In this case, the clinical diagnosis was supported by multiple converging lines of evidence, including a temporal relationship to ICI exposure, imaging features, colonoscopic and histopathological clues, exclusion of common etiologies, the presence of other suspected immune-related manifestations, and a relatively rapid response to glucocorticoid therapy. Nevertheless, because full-thickness intestinal biopsy, objective gastrointestinal motility testing, and formal autonomic function testing were not performed, the diagnosis of irIPO remained presumptive and lacked pathological or functional confirmation.

The first condition that must be distinguished from irIPO is mechanical intestinal obstruction. Because this patient had previously undergone gastric cancer surgery, postoperative adhesive obstruction or functional disturbance had to be considered (10). However, the symptoms developed several months after surgery, and imaging did not reveal a definite transition point, a fixed level of obstruction, or a focal obstructive lesion. More importantly, when conventional measures such as bowel rest and decompression produced limited benefit, the patient’s symptoms improved rapidly after glucocorticoid therapy, which is inconsistent with the usual course of uncomplicated mechanical obstruction. Tumor progression or peritoneal disease should also be considered in the differential diagnosis. In patients with malignancy, local recurrence, peritoneal metastasis, or tumor compression may all impair intestinal transit, and conventional imaging has limitations in detecting small lesions and accurately defining disease extent (11, 12). In this patient, however, PET/CT showed no clear evidence of recurrence, peritoneal implantation, or mass effect causing compression, and the clinical course was not consistent with progressively worsening malignant obstruction. In addition, the rapid improvement after immunosuppressive therapy further reduced the likelihood that tumor progression was the primary cause.

Common secondary causes of IPO, including infection, medications, and metabolic disturbances, should also be excluded systematically. Infection may not only cause diarrhea and intestinal inflammation, but may also lead to secondary intestinal paralysis in the setting of systemic inflammatory response or sepsis (13). In patients receiving ICIs, infection and immune-related inflammation are not mutually exclusive and may coexist; therefore, even when endoscopic and histopathological findings support colitis, this does not obviate the need for appropriate infectious evaluation (14, 15). In addition, opioids, anticholinergic agents, and certain sedatives may suppress intestinal motility (13, 16), while severe electrolyte disturbances, thyroid dysfunction, and hypoperfusion may also precipitate paralytic ileus (17–20). In this case, there was no clear evidence of gastrointestinal infection or opioid exposure, and key indicators, including electrolyte levels, blood glucose, lactate, and thyroid function, were insufficient to fully account for such a severe and progressive pseudo-obstructive phenotype. These findings further support the diagnosis of irIPO.

Paraneoplastic neurologic syndrome (PNS) and primary autoimmune autonomic neuropathy are also important considerations in the differential diagnosis. ICIs may trigger or exacerbate antibody-associated PNS, which can manifest as severe IPO (21–23). Cases of paraneoplastic IPO associated with anti-Hu antibodies (21) and anti-CV2/CRMP5 antibodies (24) have both been reported. Primary autoimmune autonomic neuropathy is mainly associated with anti-ganglionic acetylcholine receptor antibodies (gAChR Ab) or other autoimmune responses directed against autonomic ganglia (25). However, in many reported cases of IPO occurring in patients with cancer after ICI treatment, systematic testing for neuronal autoantibodies was not performed (26). Anti-Hu-associated enteric neuropathy often suggests irreversible damage to the enteric plexus and may be accompanied by lymphoplasmacytic infiltration (22). It typically responds poorly to glucocorticoids and is associated with severe neurologic dysfunction and high mortality (21). Moreover, autonomic neuropathy is clinically heterogeneous and lacks specific manifestations, making it easy to overlook or misdiagnose. Accordingly, evaluation for PNS remains important in patients with high-risk tumors, such as small cell lung cancer, neuroblastoma, and neuroendocrine neoplasms (27). In this case, because a full panel of neuronal autoantibodies and comprehensive autonomic function testing were not performed, PNS or primary autoimmune autonomic neuropathy could not be completely excluded. Nevertheless, given the temporal relationship and the response to glucocorticoid therapy, the findings in this case more strongly support an ICI-related acquired immune-mediated intestinal motility disorder.

Hirschsprung disease and allied disorders are generally not the primary differential diagnoses in adult patients presenting with obstruction-like symptoms. These conditions are mainly related to congenital abnormalities of enteric nervous system development; they are rare in adults and are often associated with an earlier history of chronic constipation or refractory motility disorders (28). In this case, this possibility was considered unlikely. However, if a patient has a pre-existing history of chronic intestinal dysmotility, or if full-thickness pathology demonstrates ganglion cell abnormalities, myenteric plexus hypoplasia, or acquired hypoganglionosis, the possibility that a pre-existing enteric nervous system disorder was triggered or exacerbated by ICI exposure should still be taken into account (29).

### 3.2 Pathogenesis of irIPO

Gastrointestinal motility, transit, secretion, storage, and excretory functions are governed by complex regulatory mechanisms. Intestinal peristalsis depends on coordinated interactions among the autonomic nervous system, the enteric nervous system (ENS), intestinal smooth muscle, and interstitial cells of Cajal (ICCs). Damage affecting any of these components, either alone or in combination, may result in intestinal dysmotility and ultimately lead to IPO (30, 31). The pathogenesis of irIPO remains incompletely understood. Available evidence suggests that it is unlikely to result from a single factor. Based on the clinical course, ancillary findings, and treatment response in this case, we believe that ICI-related injury to the enteric nervous system and autonomic dysfunction may constitute the main pathophysiological basis, whereas immune-related enteritis, susceptibility associated with prior gastrectomy, dysregulation of the microbiota-immune-neural axis, and abnormal ICC function may further exacerbate intestinal dysmotility and promote the development of irIPO.

One of the best-supported mechanisms underlying ICI-related IPO is immune-mediated inflammatory injury to the enteric nervous system, particularly involving the myenteric plexus. The enteric nervous system plays a central role in gastrointestinal motility and may be a direct target of immune-mediated injury (32). By releasing peripheral immune inhibition and activating effector T cells, ICIs may trigger immune-mediated attack against the ENS. Immune-mediated neuronal injury and altered neurotransmitter release may disrupt intestinal motor regulation and ultimately lead to IPO (33–35). Pathological findings reported in previous cases include CD8+ T-cell infiltration around the myenteric plexus and, in severe cases, extensive loss of enteric ganglion cells (9, 36, 37). Current neuropathological evidence supports a stage-based model of progression in which immune responses in inflammatory enteric neuropathy lead initially to neuronal dysfunction and degeneration and, over time, may progress to complete enteric neuronal loss (38). Collectively, these findings suggest that injury to the enteric nervous system may be a key mechanistic basis of irIPO.

More extensive immune-mediated autonomic dysfunction may also contribute to the pathogenesis of irIPO. ICI-related gastrointestinal autonomic dysfunction may present as autoimmune autonomic ganglionopathy or autonomic neuropathy and may be accompanied by extraintestinal autonomic manifestations, including genitourinary or cardiovascular dysfunction (7, 25, 39). Accordingly, gastrointestinal autonomic neuropathy should be strongly suspected in patients with prior ICI exposure who develop subacute severe gastrointestinal dysmotility together with autonomic or neurologic symptoms (7). Such complications may progress rapidly and require early recognition and prompt immunosuppressive treatment (40). In this case, the patient developed urinary retention during the later course of illness, and both abdominal symptoms and urinary function improved almost synchronously after methylprednisolone treatment. This clinical pattern supports the possibility that the underlying pathological process was not confined to localized intestinal inflammation but also involved broader immune-mediated autonomic dysfunction.

ICI-colitis may have been an important contributing factor in this case. Persistent mucosal inflammation and the local release of inflammatory mediators may disrupt enteric neuromuscular regulation and thereby aggravate intestinal dysmotility. Previous reports have described ICI-associated colitis accompanied by obstruction-like symptoms that improved after glucocorticoid treatment once mechanical obstruction had been excluded (41, 42). In addition, evidence from inflammatory bowel disease suggests that intestinal inflammation may alter enteric neuronal excitability, inhibitory neural signaling, and enteric glial function, thereby impairing gastrointestinal motility (43). In this case, colonoscopic and histopathological findings supported a diagnosis of ICI-colitis. However, mucosal inflammation alone was unlikely to fully explain the marked bowel dilatation and severe dysmotility observed. Rather, colitis may have acted as a contributing factor alongside ICI-related enteric neuropathy and/or autonomic dysfunction.

The post-gastrectomy state may have provided a predisposing background for the development of irIPO in this case. Previous studies have suggested that, in addition to anatomical alterations, gastrectomy may disrupt local neural regulatory networks, impair ICC networks, and remodel the intestinal microbiota, thereby weakening gastrointestinal neuromuscular regulation and increasing susceptibility to dysmotility (10, 44, 45). Alterations in the intestinal microbiota may further influence gastrointestinal motility through interactions with the immune system, enteric nervous system, and autonomic nervous system (46–48). Given that ICI therapy itself has been associated with dysbiosis and susceptibility to irAEs, microbiota-related immune activation may have further aggravated local inflammation and disrupted intestinal neuromuscular homeostasis in this setting (49–52). ICCs are responsible for generating and propagating gastrointestinal slow-wave activity and are essential for coordinated intestinal motility; their dysfunction has been associated with a range of gastrointestinal motility disorders (53, 54). Experimental and clinical observations in ICI-related delayed gastric emptying further suggest that immune activation may impair ICC function through macrophage dysregulation, reduced heme oxygenase-1 expression, oxidative stress, and inflammatory injury (55). A myogenic mechanism may also have contributed, as ICI-related myositis has been reported in association with gastrointestinal or esophageal motility abnormalities (56). However, direct evidence linking the post-gastrectomy state, dysbiosis, ICC dysfunction, or myogenic injury to irIPO remains limited, and these mechanisms should still be regarded as exploratory hypotheses.

The mechanisms described above are not entirely independent; rather, they may be interrelated and collectively contribute to the development and progression of irIPO. This complex pathophysiological interplay makes the diagnosis and management of irIPO particularly challenging. Because comprehensive functional, pathological, and neuroimmunological evidence was unavailable, it was not possible to determine with certainty whether the predominant lesion primarily involved the enteric nervous system, intestinal smooth muscle, or interstitial cells of Cajal, or instead reflected secondary involvement of deeper neuromuscular structures caused by local inflammation. Importantly, incomplete diagnostic evidence is common in routine clinical practice in such cases. Invasive or specialized investigations may not be feasible because of disease severity, limited patient tolerance, or restricted availability of diagnostic resources. Therefore, the mechanistic discussion presented in this report is based mainly on the available literature and integrated clinical inference, and further validation will require additional clinical evidence as well as more systematic studies incorporating motility testing, pathological assessment, and neuroimmunological assessment.

### 3.3 Clinical identification and management of irIPO

**Figure 4:**
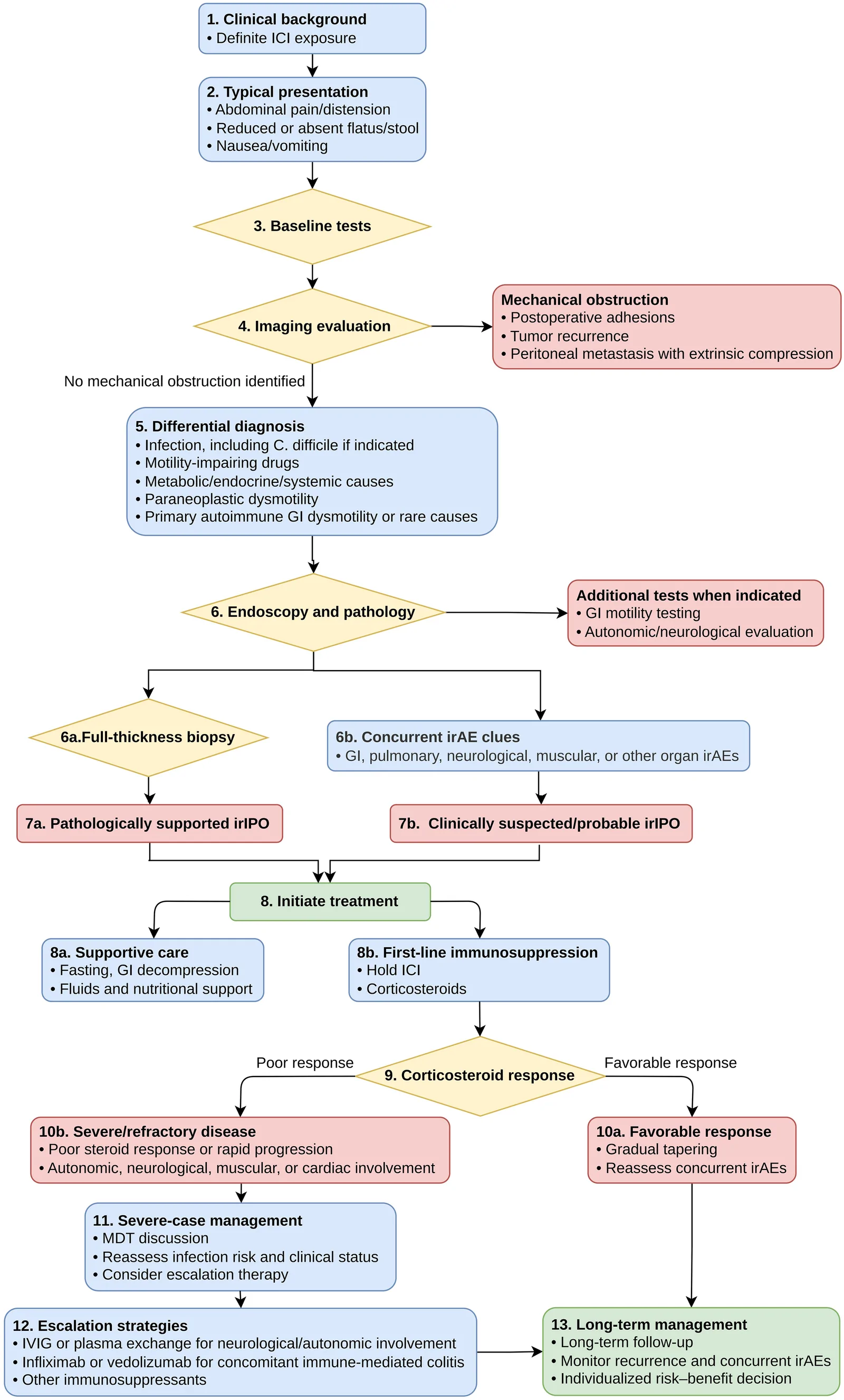
Proposed diagnostic approach for suspected irIPO, including clinical evaluation, differential diagnosis, and multidisciplinary assessment. Abbreviations: GI, gastrointestinal; ICI, immune checkpoint inhibitor; irAE, immune-related adverse event; irIPO, immune checkpoint inhibitor-related intestinal pseudo-obstruction; IVIG, intravenous immunoglobulin; MDT, multidisciplinary team.

In a previous review of 21 cases of gastrointestinal autonomic dysfunction following immunotherapy, the most common primary malignancies were melanoma and lung malignancies (7). By contrast, reports of irIPO in patients with gastric cancer are even rarer, making the present case particularly relevant to clinical practice. In postoperative patients with gastric cancer, symptoms such as abdominal distension, abdominal pain, constipation, nausea, and vomiting are easily attributed to postoperative adhesions, tumor progression, infection, or nonspecific ileus, which may delay recognition of an immune-related etiology and postpone the initiation of immunosuppressive therapy. Accordingly, irIPO should be regarded as a high-risk irAE requiring early recognition and timely intervention. Real-world data further reinforce this clinical concern. A FAERS database study showed that the overall median time to onset of ICI-related intestinal obstruction was 36 days, with most cases occurring within the first 2 months after treatment initiation; approximately 74% of cases required treatment discontinuation, and some had serious outcomes (8). These findings suggest that clinicians should maintain a high level of vigilance for persistent manifestations of gastrointestinal dysmotility even in the early phase of ICI treatment, rather than dismissing them as nonspecific manifestations of common postoperative complications or tumor progression. Based on the diagnostic challenges illustrated by this case and previously reported cases, we propose a diagnostic approach to the evaluation of suspected ICI-related IPO (**Figure 4**).

Clinically, irIPO should be considered when a patient develops persistent constipation, abdominal distension, abdominal pain, nausea, vomiting, or reduced passage of flatus and stool after ICI exposure, especially when imaging fails to show clear evidence of mechanical obstruction. Some patients may also present with broader manifestations of autonomic dysfunction, such as dysphagia. Early recognition of irIPO is crucial to avoiding unnecessary surgical intervention. If other organ-specific irAEs are present simultaneously, particularly clues suggesting neurologic involvement, autonomic dysfunction, or myositis, clinicians should be even more alert to the possibility of immune-related autonomic involvement (39).

From a diagnostic perspective, the evaluation of irIPO should follow the principle of excluding common causes first and then integrating the immune-related context. First, mechanical obstruction should be systematically ruled out, including postoperative adhesions, recurrent tumor, peritoneal metastasis, and local compressive lesions. Second, infectious causes should be assessed, particularly in patients with diarrhea and inflammatory features, in whom microbiological workup should be performed as comprehensively as possible. Third, drug-induced and metabolic causes of ileus should also be evaluated in parallel, including opioid exposure, electrolyte disturbances, endocrine abnormalities, and severe systemic illness. When feasible, paraneoplastic syndromes and primary autoimmune gastrointestinal motility disorders should also be taken into consideration. In clinically complex cases, the final diagnosis often depends on integrated clinical judgment after common causes have been systematically excluded.

With regard to ancillary testing, plain abdominal radiography may serve as an initial screening tool in patients with suspected chronic intestinal obstruction, with common findings including dilated bowel loops and air-fluid levels. Contrast-enhanced CT or MRI is useful for identifying intraluminal and extraluminal causes of mechanical obstruction and is an important imaging modality for evaluating small-bowel lesions. Upper gastrointestinal endoscopy and colonoscopy are helpful not only for detecting mechanical lesions that may be missed on imaging, but also for excluding mucosal inflammation or other organic lesions through biopsy (57). Pathological evaluation of the myenteric plexus or full-thickness intestinal biopsy may provide important diagnostic support; however, such specimens are often difficult to obtain in routine practice. Therefore, in most cases, the diagnosis still relies on exclusion and integrated clinical judgment after completion of imaging, endoscopic, and laboratory evaluation, with motility studies added when necessary.

From a therapeutic perspective, in addition to supportive measures such as bowel rest, decompression, fluid replacement, and nutritional support, careful attention should be paid to the timing of immunosuppressive therapy. Previous case reports suggest that some patients with irIPO may require earlier and more intensive immunosuppressive therapy than is typically used for conventional gastrointestinal irAEs (39, 58, 59). Some investigators have proposed that early initiation of high-dose immunosuppressive therapy may help suppress inflammatory responses within the enteric nervous system and thereby reduce the risk of irreversible neuronal injury (60). Mechanistically, glucocorticoids can suppress inflammation but cannot reverse established neuronal injury or tissue necrosis. This may partly explain why some patients respond poorly to steroid therapy and further underscores the importance of early recognition and prompt intervention. In this case, the patient showed a rapid clinical response to glucocorticoids after conservative treatment had provided limited benefit, which supports the clinical importance of early recognition and timely initiation of immunosuppressive therapy. The case reported by Qian et al. similarly suggested that prompt recognition of an immune-related etiology and early glucocorticoid treatment after the onset of initial symptoms such as constipation and abdominal distension may facilitate symptom improvement within a relatively short period (42).

For patients who respond poorly to glucocorticoids or have rapidly progressive disease, escalation of immunosuppressive therapy may be considered, although the current evidence remains limited. If corticosteroid treatment is ineffective or symptoms fail to improve, and autonomic nervous system involvement or severe disease is suspected, plasma exchange (PLEX) or intravenous immunoglobulin (IVIg) may be considered in accordance with management principles for neurologic irAEs (61, 62). The immunosuppressive agents infliximab and vedolizumab have demonstrated favorable efficacy and safety in inflammatory bowel disease and ICI colitis (63, 64), but experience with their use in irIPO remains sparse and is insufficient to support evidence-based recommendations. Available reports suggest that vedolizumab may have limited efficacy in some cases of steroid-refractory irIPO, while infliximab has been used only in isolated case reports (9, 65). After treatment failure, some patients have required colostomy or maintenance support with parenteral nutrition, and both quality of life and clinical outcomes have generally been poor. Therefore, in patients with severe manifestations such as marked autonomic dysfunction, neurologic involvement, myositis, or myocarditis, multidisciplinary collaboration should be initiated as early as possible, involving oncology, gastroenterology, neurology, nutrition, and, when necessary, surgery. The decision to escalate immunosuppressive therapy should be individualized after comprehensive consideration of infection risk, the extent of organ involvement, and the patient’s overall condition.

Previous reports have suggested that symptoms may recur or worsen after re-exposure to ICIs, sometimes necessitating more intensive immunosuppressive treatment (7). Therefore, ICI rechallenge should be approached with extreme caution and individualized according to the severity of prior toxicity, the anticipated oncologic benefit, and the availability of alternative treatment options. Given that gastrointestinal irAEs may follow a prolonged or relapsing course, long-term follow-up and management remain necessary even after symptom resolution in order to monitor for recurrence and assess nutritional recovery.

## 4 Conclusion

This report describes a case of immune checkpoint inhibitor-related intestinal pseudo-obstruction (irIPO) with multisystem irAEs in a post-gastrectomy patient with gastric cancer during adjuvant nivolumab therapy. Although irIPO is rare, it may occur as part of a broader spectrum of multisystem irAEs. In patients who develop obstruction-like symptoms after ICI treatment without evidence of mechanical obstruction, an immune-mediated etiology should be considered early, and active screening for other irAEs should be undertaken. Early recognition, integration of imaging and pathological findings, close multidisciplinary collaboration, and timely initiation of glucocorticoid therapy may improve clinical outcomes. Further case accumulation and mechanistic studies are needed to clarify the pathogenesis of irIPO and to optimize diagnostic approaches, risk stratification, and treatment strategies.

## Data Availability

The raw data supporting the conclusions of this article will be made available by the authors, without undue reservation.
